# Dexmedetomidine alleviates insulin resistance in hepatocytes by reducing endoplasmic reticulum stress

**DOI:** 10.1007/s12020-019-02118-1

**Published:** 2019-11-02

**Authors:** Fanfan Liu, Shaojun Zhu, Lifeng Ni, Ling’er Huang, Kuirong Wang, Yanfeng Zhou

**Affiliations:** Department of Anesthesiology, The First Affiliated Hospital, College of Medicine, Zhejiang University, Hangzhou, Zhejiang PR China

**Keywords:** Dexmedetomidine, Insulin resistance, Endoplasmic reticulum stress, Hepatocytes, AKT

## Abstract

**Purpose:**

Dexmedetomidine (DEX) stabilizes intraoperative blood glucose levels and reduces insulin resistance (IR), a common perioperative complication. However, the molecular mechanisms underlying these effects remain unclear. Since endoplasmic reticulum stress (ERS) is a mechanism of IR, this study sought to examine whether DEX can effectively alleviate IR by reducing ERS.

**Methods:**

HepG2 and LO2 cells were treated with different concentrations of insulin. The glucose content assay and Cell Counting Kit-8 (CCK-8) were then employed to determine the optimal insulin concentration capable of inducing IR without affecting cell viability. Insulin-resistant hepatocytes were cultured with different concentrations of DEX for 24 h, and the glucose concentration in the supernatant was measured. ERS was assessed by qPCR and western blotting. The latter was also used to quantify the expression of phosphorylated protein kinase B (p-AKT), phosphoenolpyruvate carboxykinase (PEPCK), and glucose 6 phosphatase (G6Pase), which are key proteins involved in the action of insulin.

**Results:**

After 48-h of culturing with 10 μg/mL insulin, glucose consumption in hepatocytes was found to be reduced. IR hepatocytes cultured with 10, 100, or 1000 ng/ml DEX for 24 h showed a concentration-dependent increase in glucose consumption. Elevated mRNA and protein levels of ERS markers binding immunoglobulin protein (BIP) and ER protein 29 (ERp29), were reversed by DEX treatment. Moreover, reduced p-AKT and increased PEPCK and G6Pase protein levels in IR hepatocytes were also restored following DEX treatment.

**Conclusion:**

DEX may alleviate IR in hepatocytes by reducing ERS serving to restore insulin action via the IRS-1/PI3K/AKT pathway.

## Introduction

Insulin resistance (IR) results from impaired glucose metabolism, and is characterized by a decreased sensitivity of target organs to insulin. IR is one of the most common and serious perioperative complications and is frequently associated with a longer hospital stay, increased susceptibility to infection, and higher risk of mortality [1–3].

The liver plays an important role in glucose metabolism, although adipose tissue and skeletal muscle are also targeted by insulin. IR in the liver can lead to increased gluconeogenesis and glycogen output, resulting in fasting hyperglycemia and hyperinsulinemia. On the other hand, fat mobilization and fatty acid oxidation are inhibited by insulin, and the consequent elevation in free fatty acid levels can act on insulin signaling pathways in hepatocytes to aggravate hepatic IR [4–6].

Endoplasmic reticulum stress (ERS) is an important mechanism of IR [7] involving protein kinase-like ER kinase (PERK), activating transcription factor (ATF) 6, and inositol-requiring enzyme (IRE) 1α, which are maintained in an inactive state in the endoplasm by glucose regulated protein 78 (also known as binding immunoglobulin protein [BIP]) under normal conditions. Under conditions of hypoxia or excess sugar, the number of misfolded proteins increases; the above proteins dissociate from BIP and are activated, causing c-Jun N-terminal kinase-regulated insulin receptor substrate phosphorylation and in extreme cases, apoptosis through the CCAAT/enhancer-binding protein homologous protein pathway [8]. The insulin receptor substrate-1/phosphatidylinositol 3-kinase/protein kinase B (IRS-1/PI3K/AKT) pathway is a classical pathway for insulin action. Tyrosine phosphorylation of IRS-1 and binding to PI3K promote AKT activation, mainly at Thr308 and Ser473 sites [9]. It has been confirmed that ERS eventually inhibits the IRS-1/PI3K/AKT signaling pathway to inhibit insulin action in liver cells [8]. PEPCK and G6Pase, downstream effectors of the IRS-1/PI3K/AKT pathway, are key enzymes for gluconeogenesis and glycogenolysis respectively, and are directly related to changes in blood glucose [10].

Dexmedetomidine (DEX), a novel α2 adrenergic agonist with sedative, analgesic, anti-inflammatory, and organ-protective effects, is widely used in anesthesia and intensive care [11–13]. DEX can maintain blood glucose stability and reduce blood glucose levels, which may be associated with the suppression of systemic inflammation and pain and the regulation of humoral immunity and complement function [13]. In burn and ischemia–reperfusion models, DEX has been shown to protect organ function by reducing ERS levels [14, 15]. Based on these findings, we speculated that DEX can stabilize blood glucose and relieve IR by reducing ERS and promoting the conduction of the IRS-1/PI3K/AKT pathway in the liver.

## Materials and methods

### Cell lines and culture

Human HepG2 and LO2 hepatoma cell lines were provided by the Research Group of Hepatobiliary Surgery, Key Laboratory of Organ Transplantation of Zhejiang Provence, China. The cells were grown in minimal Eagle’s medium (MEM; Gino Bio, Hangzhou, China) and Dulbecco’s modified Eagle’s medium (DMEM; Gino Bio, Hangzhou, China), respectively, supplemented with 10% fetal bovine serum (Wisent, Saint-Jean-Baptiste, QC, Canada) and 1% penicillin–streptomycin in a 5% CO_2_ atmosphere at 37 °C. Cells were used for experiments when they reached 90% confluence.

### Cell viability assay

Cell viability was evaluated with a Cell Counting Kit (CCK)-8 (Dojindo Laboratories, Kumamoto, Japan). Approximately 5 × 10^3^ HepG2 cells and 1 × 10^4^ LO2 cells were seeded in each well of a 96-well plate. Medium containing 10% CCK-8 reagent was added to the well, followed by incubation for 1–3 h at 37 °C. When the color of the medium turned dark orange, optical density at 450 nm (OD_450_) was measured and a standard curve of cell viability was generated [16].

### Analysis of glucose content

The glucose content of the medium was measured by the glucose oxidase-peroxidase method using a commercial kit (Rsbio, Shanghai, China). HepG2 and LO2 cells were seeded as described above and cultured in the presence of insulin (Sigma-Aldrich, St. Louis, MO, USA) or DEX (Jiangsu Hengrui Medicine Co., Lianyungang, China). The medium was changed to serum- and Phenol Red-free DMEM or MEM (Wisent) for 12 h, and the mixture of R1 and R2 in the kit was added to the supernatant at a ratio of 1:200 according to the instructions, followed by incubation at 37 °C for 15 min. OD_505_ was measured and a standard curve of glucose content was generated. Glucose consumption was calculated as the difference in the glucose contents of experimental and blank wells [17, 18].

### Model of IR

Approximately 5 × 10^3^ HepG2 cells and 1 × 10^4^ LO2 cells were seeded in each well of a 96-well plate. When the cells were attached, medium containing different concentrations of insulin (0, 1, 2.5, 5, 10, and 20 μg/mL) was added and the cells were cultured for 24, 48, and 72 h [17, 19, 20]. The insulin concentration that caused the largest decrease in glucose consumption without affecting cell viability was selected as the IR model.

### Quantitative real-time (qRT-) PCR

BIP and ER protein (ERp)29 mRNA levels were evaluated by qRT-PCR. Cells were washed with phosphate-buffered saline (PBS) and the total RNA was extracted using TRIzol reagent (Solar Bio, Beijing, China); cDNA was synthesized with the PrimeScript RT Reagent Kit with gDNA Eraser (Takara Bio, Otsu, Japan), and qRT-PCR was performed using SYBR Premix Ex Taq II (Tli RNaseH Plus; Takara Bio) under the following conditions: 95 °C for 30 s, followed by 40 cycles of 95 °C for 5 s and 60 °C for 30 s. The following forward and reverse primers were used: ERp29, 5′-CCTGGATACGGTCACTTTCTACA-3′ and 5′-AGTTTTCAGCAAGACGCTTGA-3′; BIP, 5′-GAGATCATCGCCAACGATCAG-3′ and 5′-ACTTGATGTCCTGCTGCACAG-3′; and glyceraldehyde 3-phosphate dehydrogenase (control), 5′-ACTTTGGTATCGTGGAAGGACTCAT-3′ and R:5′-GTTTTTCTAGACGGCAGGTCAGG-3′. The relative expression levels of BIP and ERp29 were determined with the comparative cycle threshold (2^−ΔΔCt^) method.

### Western blot analysis

After two washes with PBS, cells were lysed with buffer containing 2% phenylmethylsulfonyl fluoride and 1% protease inhibitor cocktail. Protein concentration was measured with a bicinchoninic acid assay kit. Equal amounts of protein (~30 μg) were separated by 10% sodium dodecyl sulfate–polyacrylamide gel electrophoresis and transferred to a polyvinylidene difluoride membrane (Merck Millipore, Billerica, MA, USA) for ~100 min. After blocking with 5% milk, the membrane was incubated overnight at 4 °C in a 1.25% milk solution containing primary antibodies including anti-BIP, anti-AKT and anti-pAKT, (1:1000; Cell Signaling Technology, Danvers, MA, USA; Cat: #3183; #4060; #4691), ERp29 (1:2000) and G6Pase, (1:500; Abcam, Cambridge, MA, USA; Cat:ab176573; ab83690), PEPCK, (1:2000; Proteintech Group, Chicago, IL, USA; Cat:16754-1-AP). The membrane was washed thrice with Tris-buffered saline containing 0.1% Tween-20, and incubated for 2 h at room temperature with a 1.25% milk solution containing horseradish peroxidase-conjugated goat anti-rabbit IgG (1:2000; Beyotime Institute of Biotechnology, Shanghai, China; Cat:A0208). Protein bands were developed by enhanced chemiluminescence (Fude Bio, Hangzhou, China) and signal intensity was determined using Image Lab software (Bio-Rad, Hercules, CA, USA).

### Statistical analysis

Data were obtained from at least three independent experiments and are reported as the mean ± SD. Statistical analyses were performed using SPSS v.13.0 (Chicago, IL, USA). Analysis of variance was used for multiple group comparisons. Statistical significance was set at *P* < 0.05.

## Results

### Establishment of the hepatic IR model

**Fig. 1:**
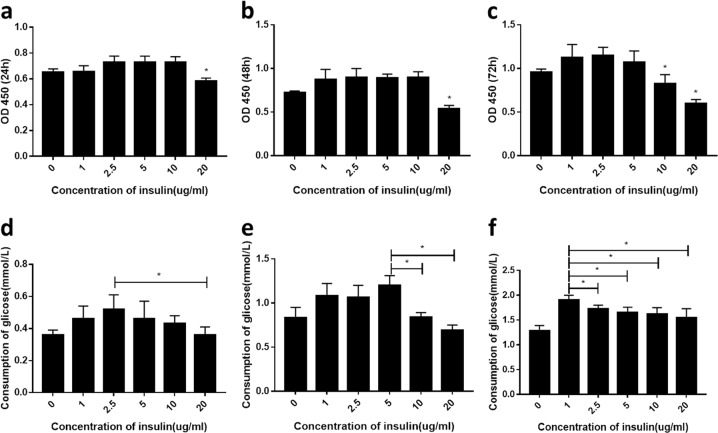
Media containing different concentrations of insulin affect viability and glucose consumption in HepG2 cells (**a**–**f**), cell viability (**a**–**c**), and glucose consumption (**d**–**f**) at 24 h (**a**, **d**), 48 h (**b**, **e**), and 72 h (**c**, **f**). A representative histogram from three individual experiments is shown, and data are presented as mean ± SD. *P < 0.05 vs. blank group

**Fig. 2:**
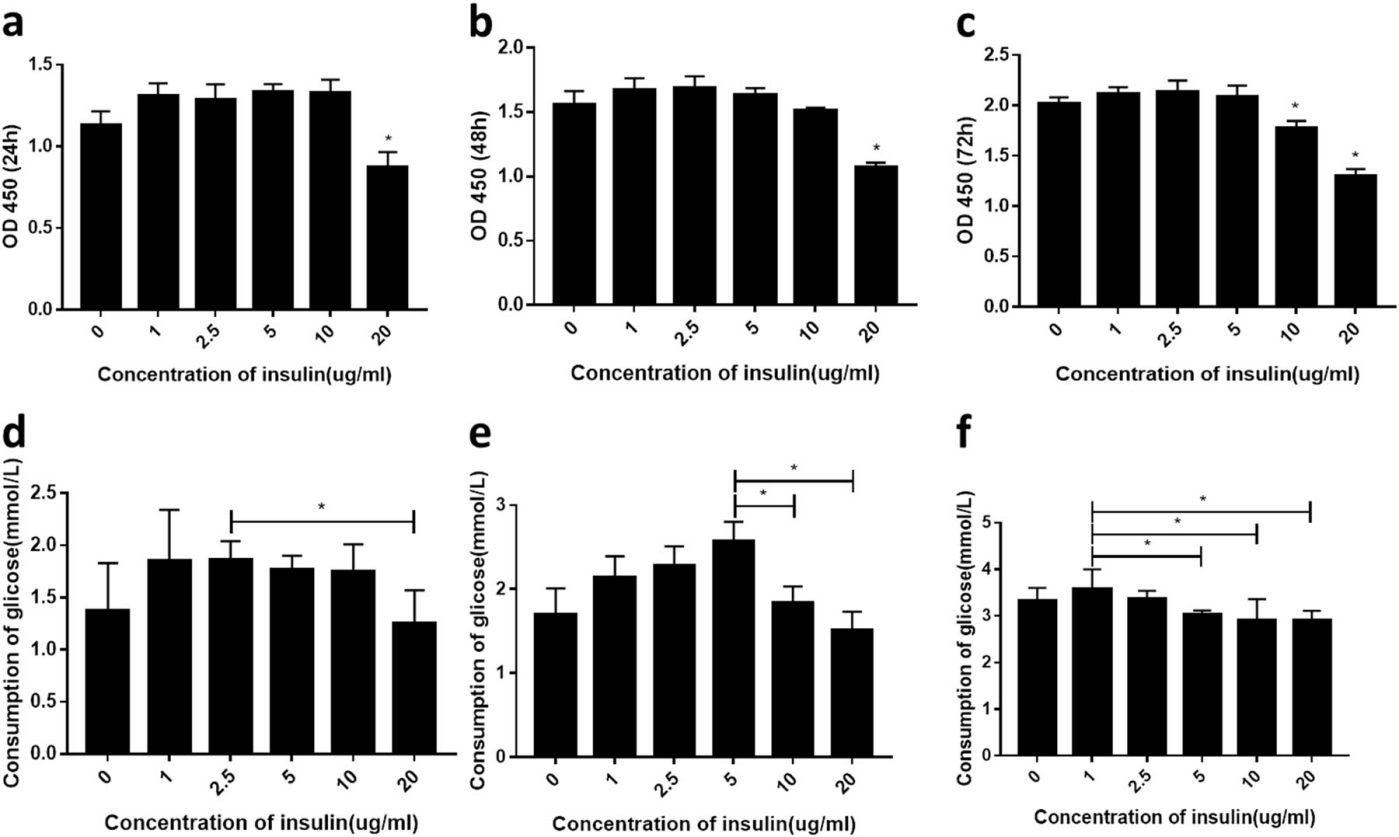
Media containing different concentrations of insulin affect viability and glucose consumption in LO2 cells (**a**–**f**), cell viability (**a**–**c**), and glucose consumption (**d**–**f**) at 24 h (**a**, **d**), 48 h (**b**, **e**), and 72 h (**c**, **f**). A representative histogram from three individual experiments is shown, and data are presented as mean ± SD. *P < 0.05 vs. blank group

In order to establish a model of hepatic IR, HepG2, and LO2 cells were incubated in a medium containing different concentrations of insulin (0, 1, 2.5, 5, 10, and 20 μg/mL) for 24, 48, and 72 h. Treatment with 20 μg/ml insulin for 24 h markedly reduced cell viability compared with the blank control group (*P* < 0.05; Figs 1 and 2). On the other hand, treatment with 10 μg/mL insulin for 72 h reduced cellular activity relative to the control cells (*P* < 0.05), as evidenced by the decrease in glucose consumption, but had no effect on cell viability within the first 48 h. The greatest decreases in glucose consumption were observed with 10 μg/mL insulin treatment for 48 h and 5 μg/mL insulin treatment for 72 h (*P* < 0.05), indicating that cells in these groups showed the strongest IR. However, cells cultured in the presence of insulin for 72 h appeared unhealthy and the culture medium was cloudy. We therefore used 10 μg/mL insulin treatment for 48 h as the conditions to generate the IR model.

### DEX has no effect on HepG2 and LO2 cell viability

To exclude the effect of reduced cell viability on glucose consumption, we cultured HepG2 and LO2 cells with different concentrations of DEX (10, 100, and 1000 ng/mL) [21, 22] and evaluated cell viability. There were no differences in viability between DEX-treated and control cells (*P* > 0.05; Table 1), indicating that DEX is not toxic to hepatocytes.

**Table 1:** Effects of DEX concentration on the viability of HepG2 and LO2 cells

Concentration of DEX (ng/ml)		0	10	100	1000
OD_450_	HepG2	0.795 ± 0.041	0.849 ± 0.027	0.837 ± 0.094	0.825 ± 0.046
	LO2	1.317 ± 0.044	1.287 ± 0.062	1.315 ± 0.067	1.310 ± 0.115

Data are shown as mean ± SD. Viability did not differ relative to the blank group

### DEX alleviates IR in a concentration-dependent manner

We tested the stability of the hepatic IR model and found no difference in glucose consumption in the first 24 h relative to the IR group. However, when cells were cultured in the medium containing DEX, glucose consumption was increased compared with that in the 24-h IR group (*P* < 0.05; Table 2). We also observed that glucose consumption increased with DEX concentration, suggesting that DEX alleviates IR in a concentration-dependent manner.

**Table 2:** Effects of DEX concentration on glucose consumption and stability of hepatocytes

Group		IR	IR-24 h	Concentration of DEX (ng/ml)		
				10	100	1000
Consumption of glucose (mmol/L)	HepG2	0.91 ± 0.06	0.93 ± 0.16	1.10 ± 0.02*	1.29 ± 0.13*	1.32 ± 0.13*
	LO2	2.79 ± 0.31	2.76 ± 0.20	3.52 ± 0.15*	3.65 ± 0.17*	3.98 ± 0.18*

Data are shown as mean ± SD. *P < 0.05 vs. IR group

### DEX alleviates IR by inhibiting BIP and ERp29 expression

**Fig. 3:**
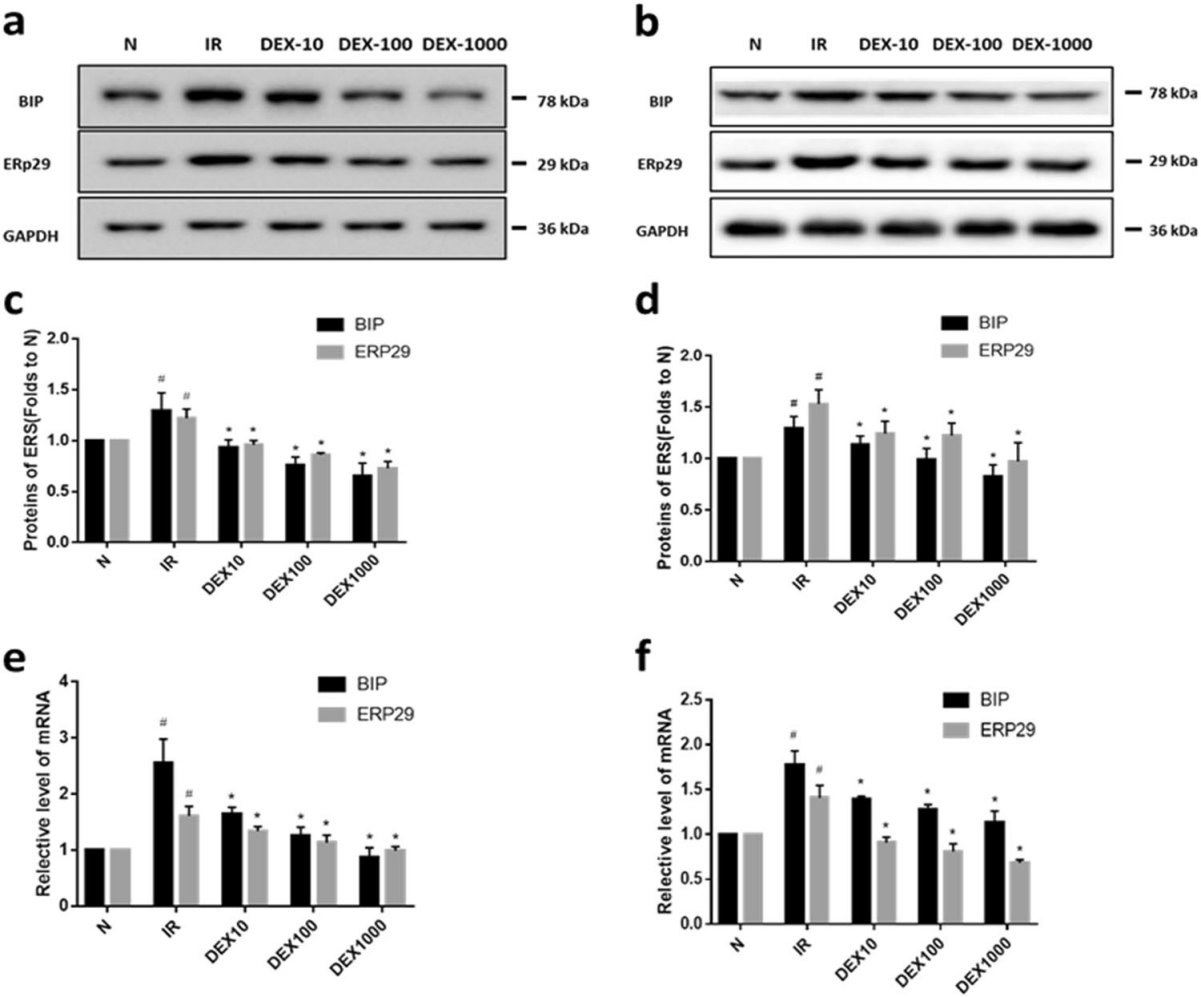
DEX reduces the expression of ERS proteins BIP and ERp29 in HepG2 and LO2 cells (**a**–**f**). The levels of protein (**a**–**d**) and mRNA (**e**, **f**) in HepG2 cells (**a**, **c** and **e**) and LO2 cells (**b**, **d** and **f**). A representative histogram from three individual experiments is shown, and data are presented as mean ± SD. ^#^*P* < 0.05 vs. normal (N) group; *P < 0.05 vs. IR group

BIP and ERp29 are markers of ERS; the mRNA and protein levels of the two markers were higher in the IR group than in the control group (*P* < 0.05; Fig. 3), suggesting that IR is closely related to ERS. However, both markers were downregulated in the DEX group compared to that in the IR group (*P* < 0.05), indicating that DEX reduced insulin-associated ERS in hepatocytes.

### DEX restores the reduced phosphorylation of AKT

**Fig. 4:**
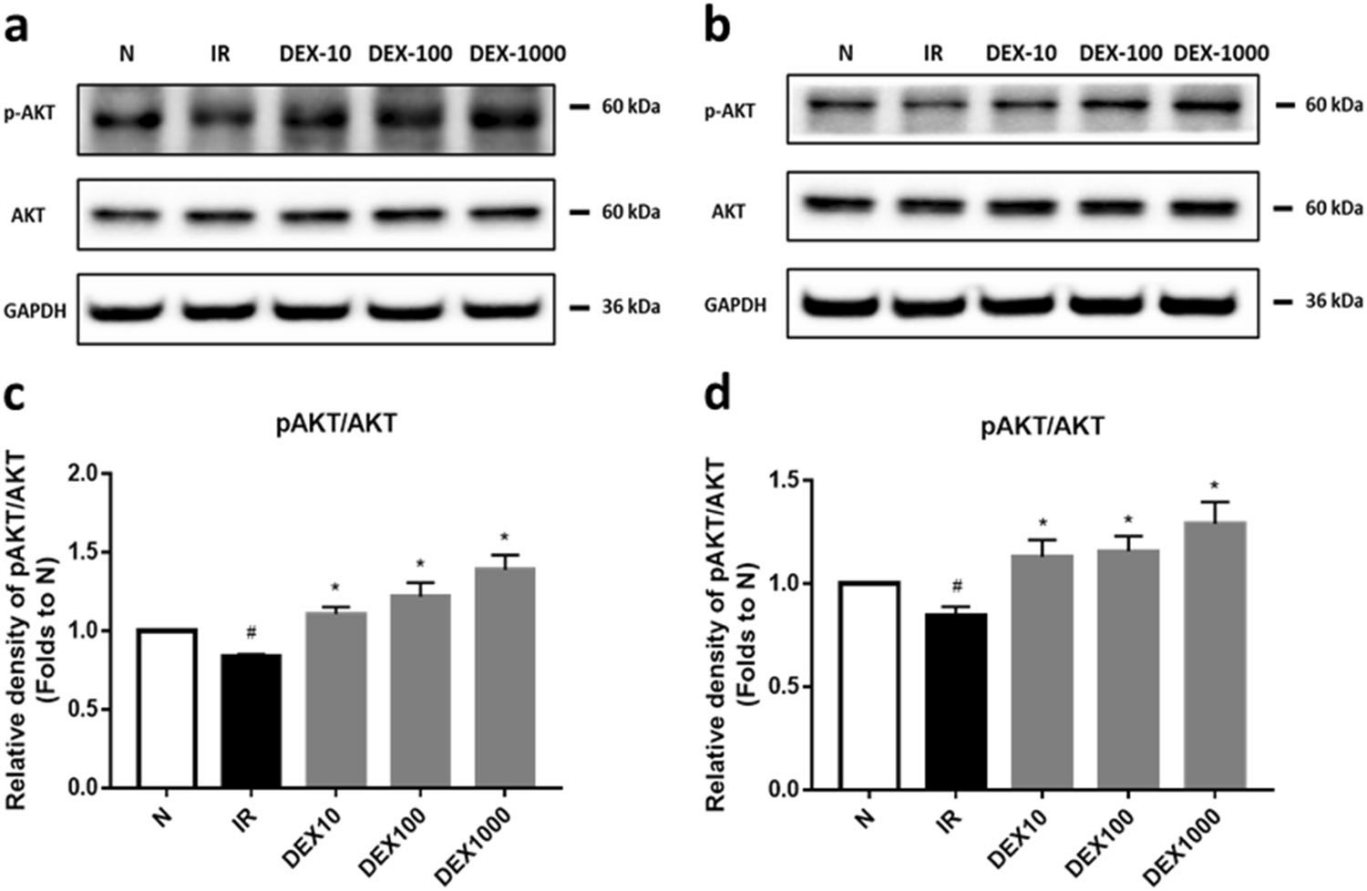
DEX restores the reduced phosphorylation of AKT in HepG2 and LO2 cells (**a**–**d**). The relative levels of protein of pAKT/AKT in HepG2 cells (**a**, **c**) and LO2 cells (**b**, **d**). A representative histogram from three individual experiments is shown, and data are presented as mean ± SD. ^#^*P* < 0.05 vs. normal (N) group; *P < 0.05 vs. IR group

To investigate whether DEX restores insulin action by reducing ERS, AKT, and p-AKT were detected by western blot. High concentrations of insulin significantly decreased insulin-stimulated phosphorylation of AKT at serine 473 (*P* < 0.05; Fig. 4), which is an important event in the insulin signaling pathway. DEX treatment restored this change and significantly increased serine phosphorylation of AKT (*P* < 0.05; Fig. 4). Hence, it is suggested that DEX may restore insulin action via the IRS-1/PI3K/AKT pathway.

### DEX restores increased gluconeogenesis and glycogenolysis

**Fig. 5:**
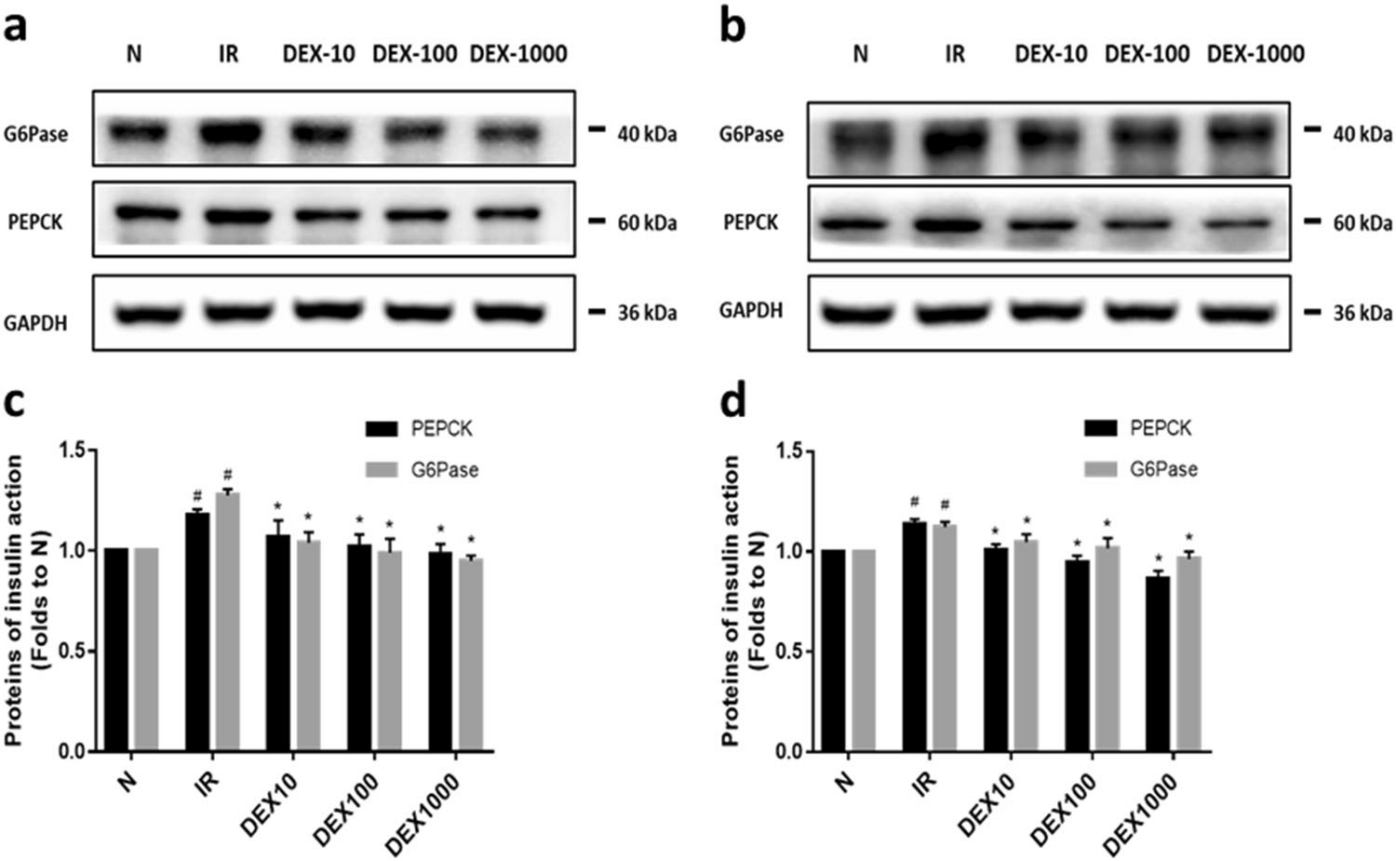
DEX restores the increased PEPCK and G6Pase in HepG2 and LO2 cells (**a**–**d**) The relative expression levels of PEPCK and G6Pase proteins in HepG2 cells (**a**, **c**) and LO2 cells (**b**, **d**). A representative histogram is from three individual experiments. Data are presented as mean ± SD. ^#^*P* < 0.05 vs. normal (N) group; *P < 0.05 vs. IR group

Gluconeogenesis and glycogenolysis are important mechanisms associated with glucose metabolism in the liver; with PEPCK and G6Pase acting as key enzymes within these pathways, respectively. The expression of PEPCK and G6Pase proteins was significantly increased (*P* < 0.05; Fig. 5) in high insulin-induced IR hepatocytes, however, they were found to be significantly decreased (*P* < 0.05; Fig. 5) following treatment with different concentrations of DEX. This indicates that DEX may eventually reduce gluconeogenesis and glycogenolysis in the liver, thereby serving to maintain blood glucose stability.

## Discussion

In this study, we showed that IR is associated with ERS. Treatment of IR hepatocytes with DEX increased glucose consumption, implying that DEX can alleviate IR; this was accompanied by downregulation of BIP and ERp29. These results indicate that DEX alleviates hepatic IR by decreasing ERS via suppression of the ERS proteins BIP and ERp29. Meanwhile, the increase in p-AKT and reduction in PEPCK and G6Pase also indicate that DEX may restore insulin activity through the IRS-1/PI3K/AKT pathway while inhibiting gluconeogenesis and glycogenolysis.

HepG2 cells are derived from human hepatocarcinoma tissue, which secretes a variety of plasma proteins, including albumin, α2-macroglobulin, plasminogen, and iron transfer protein; these cells are similar to normal human hepatocytes in terms of their biochemical characteristics and biosynthetic capacities [23]. The liver synthesizes, stores, and releases glycogen, which is important for glucose metabolism. Therefore, HepG2 cells are an ideal cell model for investigating the mechanisms of IR and testing the efficacy of hypoglycemic drugs [24, 25]. We also used LO2 cells, which are normal hepatocytes, for further validation in this study.

In vitro IR models are mainly generated in hepatocytes, fat cells, and skeletal muscle cells using high concentrations of insulin; medium containing high levels of sugar and fat; dexamethasone; palmitic acid; and tunicamycin [26–29], although a high insulin concentration is most frequently used [30]. Dexamethasone, a kind of glucocorticoid, is easily confused with dexmedetomidine (owing to similar abbreviations), which induces IR by inhibiting the binding of insulin to the insulin receptor. In this study, we determined that an insulin concentration of 10 μg/ml applied to HepG2 and LO2 cells for 48 h resulted in the lowest glucose consumption while cell viability was unaffected. In previous studies, glucose consumption in normal cells was used as the control [24, 25], but we found that insulin has a mitogenic effect on hepatocytes, although there were no changes in proliferation rate within a certain concentration range. This has also been described by other investigators [31, 32]. Since cell proliferation affects glucose consumption, we compared cells with similar proliferation rates but selected those with the highest glucose consumption within each group as the control; IR was identified based on a decrease in glucose consumption. The group showing the largest difference in glucose consumption was considered the optimal model. We believe that this method is more objective than the approaches used in previous studies.

The three major ERS proteins are PERK, ATF6, and IRE1α; these are normally bound by BIP and maintained in an inactive state, and dissociate from BIP upon ERS to activate downstream effectors. Thus, intracellular BIP level can serve as a marker of ERS [6, 8]. ERp29 is a soluble protein located on the endoplasmic reticulum membrane [33]; ERp29 mRNA and protein levels were found to increase with BIP expression in a tunicamycin-induced ERS model of Ins-1 islet cells; additionally, ERp29 was shown to be involved in ER stress-mediated islet cell damage [34]. The occurrence of ERS further inhibits insulin action through the IRS-1/PI3K/AKT pathway as one of the downstream mechanisms of ERS [8]. Phosphorylation of IRS-1 at serine sites and decreased phosphorylation of AKT result in disrupted insulin signaling and induction of insulin resistance. Liver-specific gluconeogenesis and glycogenolysis have a direct impact on the regulation of blood glucose, with PEPCK and G6Pase serving as particularly important factors in these two pathways [10].

Clinical studies have demonstrated that DEX stabilizes perioperative stress-induced hyperglycemia and decreases IR in patients with diabetes; this may be related to a reduction in sympathetic activity, analgesia, and anti-inflammatory response [13]. DEX exerted protective effects in lungs by reducing the levels of ERS in a model of ischemia-reperfusion injury [35], and was shown to be closely related to ERS caused by burns and glucose deprivation [12, 14, 16]. However, the effects of DEX on ERS in an IR model have not been previously reported. Our results demonstrate that DEX can stabilize blood glucose level by reducing ERS and restoring insulin action. It is known that ERS is associated with the release of inflammatory factors, which may indirectly affect nuclear factor-κB signaling [35]. Whether DEX further reduced ERS by suppressing inflammation to affect blood glucose level is unclear, since we only measured glucose consumption in the medium. Furthermore, although we observed that DEX inhibited the expression of ERS proteins, the role of ERS in IR was not confirmed by gene knockout experiments. Additional studies are also needed to clarify the role of inflammation in this process. The IRS-1/PI3K/AKT pathway in the liver is inhibited during IR. The phosphorylation of AKT is reduced, resulting in the activation of glycogen synthase kinase-3 (GSK-3), which triggers glycogen synthase (GS) phosphorylation and interferes with glycogen synthesis [36]. Although we did not directly examine the effects of DEX on GSK-3 and GS, we did observe that DEX reduced the levels of gluconeogenesis and glycogenolysis in the liver. Therefore, additional in vivo experiments are required to provide further evidence for clinical work. Nonetheless, our findings indicate that treatment with DEX may be an effective intervention for preventing perioperative IR and provide novel insights into the molecular basis for its beneficial effects.
